# Acknowledgment to Reviewers of *Pharmaceutics* in 2021

**DOI:** 10.3390/pharmaceutics14020314

**Published:** 2022-01-28

**Authors:** Pharmaceutics Editorial Office

**Affiliations:** MDPI AG, St. Alban-Anlage 66, 4052 Basel, Switzerland

Rigorous peer-reviews are the basis of high-quality academic publishing. Thanks to the great efforts of our reviewers, *Pharmaceutics* was able to maintain its standards for the high quality of its published papers. Thanks to the contribution of our reviewers, in 2021, the median time to first decision was 16 days and the median time to publication was 38 days. The editors would like to extend their gratitude and recognition to the following reviewers for their precious time and dedication, regardless of whether the papers they reviewed were finally published:
A. S. M. Monjur Al HossainKheireddine El-BoubbouAaron AlfordKi Su KimAaron HeffernanKibeom KimAbdalla H. KaroyoKirsty L. WilsonAbdel Naser ZaidKit Leong CheongAbdelbary ElhissiKi-Tae HaAbdul AhadKlemen BohincAbdul BasitKoichi KitagawaAbdullah IsrebKonstantin Yu ZhizhinAbhijit RangnekarKorzhikova-Vlakh EvgeniaAbhishek DeyKostas BethanisAbhishek JuluriKota KodamaAbshar HasanKristan WorthingtonAchim SauerKristaps JaudzemsAda W. Y. LeungKristiina WähäläAdam PacławskiKristina KantminienėAdeola O. AdebisiKristopher R. GenschmerAdoracion Gomez QuirogaKrisztina Takacs-NovakAdrián MatencioKrystyna Mojsiewicz-PieńkowskaAdriana CiurbaKrzesimir CiuraAdriano DuattiKrzysztof SzczepanowiczAdriano TroiaKuangming KuoAdrienne ScheckKuldeep K. BansalAfeesh R. UnnithanKun ZhouAfzal HussainKunn HadinotoAgata PucekKuo-Ching MeiAgnes CsiszárKuthuru SureshAgnes S. KlarKwang Suk LimAgnese GagliardiKwang-Hee SonAgnieszka ChwilkowskaKyle Holmberg ViningAgnieszka Gadomska-GajadhurKyriaki S. PafitiAgnieszka KarbownikKyriakos KachrimanisAgnieszka KierysKyung Taek OhAgnieszka LatosinskaKyung-Ho RohAgnieszka Majkowska-PilipKyung-Min LimAgnieszka MarkowskaKyung-Oh DohAgnieszka MroczekLa Mata Francisco Javier DeAgnieszka Ulatowska-JarżaLacramioara PopaAhmed A. El-GendyLaila ZikoAhmed El-FiqiLamar O. MairAhmed HassanLamiaa M. A. AliAhmed Mohamed Ali KhalilLara MassaiAhmed MostafaLarry MillerAi-Ho LiaoLászló JicsinszkyAilin WangLászló MajorosAitana TamayoLaura CatenacciAiva PlotnieceLaura D’AlfonsoAjay SharmaLaura Francés-SorianoAkif Emre TüreliLaura KassAkihiro OhiraLaura LocatelliAkira InoueLaura Marcos-ZambranoAkshaya Srikanth BhagavathulaLaura Modica De MohacAkshaya TatkeLaura Oliveira-NascimentoAlan HarperLaura PolitoAlan RichardsonLaura RombolàAlba García-RodríguezLaura SbârceaAlba GonzálezLaura TrandafirAlbert RizvanovLaura VicenteAlbin KristlLaurence LagneauxAlbino MartinsLaurent SimonAlciviadis-Constantinos CefalasLaurentiu M. PopAldona KubicaLavinia VlaiaAlejandra Gutierrez-GuerreroLeah M. JohnsonAlejandro Martínez-ÁguilaLeena KadamAlejandro ParedesLefkothea PapadopoulouAlejandro Pérez LariosLei WangAlejandro Schcolnik-CabreraLen LuytAleksandar M. VeselinovicLenuta ProfireAleksandar RaškovićLeon IslasAleksander BilewiczLeonard AtanaseAleksander MendykLeonardo BascoAleksander ShtilLeonardo V. M. De AssisAleksandr V. KhilovLeopoldo SitiaAleksandra KlimczakLeszek KadzińskiAleksandra KoraćLeticia CuboAleksandra KrólickaLi FuAleksandra M. BondžićLiane Isabel Ferreira MouraAleksandra Wisłowska-StanekLiang XuAlena SiskovaLiang-Yin KeAlessandra AdroverLibor KostkaAlessandra BoschiLidia TajberAlessandra FuscoLidija BarišićAlessandra RossiLígia C. Gomes-da-SilvaAlessandra SemenzatoLiliana S. MendonçaAlessandra StacchiottiLingxiao XieAlessandro F. MartinsLingxue KongAlessandro GranitoLingyan ShiAlessio AdamianoLionel GamarraAlessio BocediLisa RileyAlessio MalfantiLiudmyla V. GarmanchukAlexa KlettnerLiujiang SongAlexander A. SpasovLjiljana ĐekićAlexander AndrianovLong Chiau MingAlexander Batista-DuharteLoredana FrascaAlexander BerezinLoredana SerpeAlexander EweLorella PasquinucciAlexander MarinLorena UrbanelliAlexander N. ShikovLoris BuschAlexander PestovLouis AllottAlexandr Alexandrovich KolobovLu XuAlexandra GaubertLuana PerioliAlexandra M. SminkLubica DudakovaAlexandra TarabolettiLuc MaroteauxAlexei B. ChukhlovinLuca BoselliAlexey ChubarovLuca De SimoneAlexey IordanskiiLuca GallelliAlexey SokolovLuca GentilucciAlexey TeplyakovLuca PaluganAlexis RumpLucia CatucciAlfio BattiatoLucia MontenegroAlfonso Garcia-BennettLuciana ScottiAli AbdallahLuciano PirolaAli Aghazadeh HabashiLucinda BessaAli Al KaissiLucinda V. ReisAli Athab Al-kinaniLucretia UdrescuAli SeyfoddinLudmila Otilia CintezaAlicja KluczykLudmila V. PuchkovaAlicja Stachelska-WierzchowskaLudwig CardonAlireza NomaniLuigi BennardoÁlvaro MeanaLuigi CapozziAmanda H. KleinLuigi MenghiniAmani TaamalliLuigi MilellaAmber DoironLuigi SapioAmelia M. SilvaLuis Alfonso Trujillo-CayadoAmiq GazdharLuis Apaza TiconaAmit TripathiLuis Cláudio Nascimento Da SilvaAmita VaidyaLuis F. Santamaría-BabíAmmar AltemimiLuis Jesús Villarreal-GómezAmparo Sánchez NavarroLuis PadrelaAmrit PaudelLuís Pinto Da SilvaAna AlcudiaLuis RodriguezAna Brotons-CantoLuisa BracciAna CazacuLukáš KučeraAna Clara AprotosoaieŁukasz ChrzanowskiAna FigueirasLukasz KurykAna Francisca BettencourtŁukasz SobottaAna I. FernandesŁukasz SzeleszczukAna I. MatesanzLuke J. MortensenAna Isabel BarbosaLynda K. HarrisAna Isabel Fraguas-SánchezM. Azizur RahmanAna M. Carmona-RibeiroM. KhotimchenkoAna MeleroM. Gracia García-MartínAna ReyMª Nieves Piña CapóAna Sara CordeiroMaciej PrzybyłekAnandharajan RathinasabapathyMaciej StawnyAnastasiia KotlyarovaMaciej TrusiakAnbazhagan SathiyaseelanMagdalena GierszewskaAnca DinischiotuMagdalena Makarska-BialokozAnca MareMagdalene J. SeilerAnca Niculina CadinoiuMaged M. A. FoudaAnca Roseanu ConstantinescuMagimairajan Issai VananAnca-Roxana PetroviciMagnus LundborgAn-Chi TienMahmoud Al-KhrasaniAncuta JurjMahmoud M. AhmedAnđelka KovačevićMahsa DabaghAnders AxelssonMai HazekawaAndi Dian PermanaMaitraye SenAndrás BallaMaja FriščićAndré AraujoMakoto EmotoAndré ChwalibogMałgorzata Anna MarćAndré Ferreira MoreiraMalgorzata BurekAndré Luís Morais RuelaMałgorzata DołowyAndré Moreni LopesMałgorzata Geszke-MoritzAndré Rolim BabyMałgorzata JanickaAndré SchulzMałgorzata Kotula-BalakAndré Silva PimentelMałgorzata KujawskaAndrea ArsiccioMałgorzata Polz-DacewiczAndrea DoderoMałgorzata Szultka-MłyńskaAndrea RagusaMallesh KurakulaAndréanne LupienManel DhahriAndreas BückManish ShahAndreas LipphausManisha SharmaAndreas OuranidisManishkumar R. ShimpiAndreia FreitasManjunatha S. MuttigiAndrej KastrinManmeet RawatAndrew J. DingleyManomi PereraAndrew KatsifisMansoori BehzadAndrew MitchellManuel Cano LunaAndrew NaylorManuel CórdobaAndrey A. ZamyatninManuel Gutierrez-AguilarAndris DišlersManuel Guzmán-NavarroAndrzej CzyrskiManuel KaulichAndrzej PawlikManuel López-LópezAndy PickettMara MarongiuAnela IvanovaMāra PilmaneAneliya MilanovaMarc NadalAneta Ostróżka-CieślikMarcin OzarowskiAngela AbruzzoMarcio José TieraAngela Aguilar-de-LeyvaMarco A. Loza-MejíaAngela CasarilMarco Antonio Loza-MejíaAngela FaccendiniMarco BiondiAngela Yen MooreMarco ChaudAngelika E. SchniekeMarco CordaniAngelina AngelovaMarco MinaAnglada Francesc RabanalMarcos Luciano BrushiAnh NguyenMarcos Martínez-GarcíaAnicuta StoicaMarcus Tullius ScottiAnife AhmedovaMarek BrzezinskiAnil VangalaMarek DrozdzikAnil VermaMarek DroździkAnisoara CimpeanMarek FolAnita Andicsova EcksteinMarek K. DobkeAnita HafnerMarek LangnerAnke BurmesterMarek OchowiakAnke SchwarzMarek PokornyAnkit K. RochaniMaria AlbaAnkit RaiMaría Ángeles PeinadoAnna ArisMaría Ángeles Peña FernándezAnna BajekMaria C. T. CangussúAnna CalarcoMaria CarafaAnna ChoromanskaMaría Cristina AreaAnna DrabczykMaria Cristina BonferoniAnna DrzazgaMaria CristofaroAnna GiammancoMaria Da Graça Costa MiguelAnna GumieniczekMaría Dea-AyuelaAnna JagusiakMaria DigiacomoAnna Kabłak-ZiembickaMaria Ermelinda S. EusébioAnna KrupaMaria Giovanna ScioliAnna LankoffMaria Gonzalez-BejarAnna LeopoldMaria Grazia CifoneAnna M. Kurek-GóreckaMaria Grazia OrtoreAnna OgrodowczykMaría Hortigón-VinagreAnna Rita BiliaMaría Jesús Alvarez-CuberoAnna SzakielMaría Jesus GarridoAnna Tankiewicz-KwedloMaría José García-BarradoAnna V. NaumovaMaria LaptevaAnnalisa DalmoroMaria Letizia MancaAnnalisa MarucaMaria LeżańskaAnnalisa RadeghieriMaria Luisa BelliAnnarita StringaroMaría Luisa MoyáAnne AndersonMaria Luisa TorreAnne RodallecMaria Manuela GasparAnroop NairMaria ModicaAnthony D. M. CurtisMaria MolinaAnthony DelalandeMaria Natalia CalienniAnthony HarkerMaria Pia AdorniAnthony HickeyMaria Pia FerrazAnthony MukwayaMaria Teresa MascellinoAnthony TavaresMaria ZychAntje VollrathMariamena ArbitrioAntoine KichlerMarián PeciarAntoine PallandreMariana Buranelo EgeaAnton ManakhovMariana PintealaAnton V. KiselevMariana S. CastroAntonella CasiraghiMariano Francesco CaratozzoloAntonella PaladinoMariarosaria MilosoAntonella RosiMarie Pierre KrafftAntonello BarresiMarie-Christine JonesAntonello Di PaoloMarie-José GoumansAntónio AlmeidaMariia NesterkinaAntonio Dominguez-AlfaroMariia SavelevaAntonio E. Martín RengelMarija IvanovAntonio FronteraMarija V. PergalAntonio LucacchiniMarijana Zovko KončićAntonio MonariMarika PellegriniAntonio PicarelliMarika RuponenAntonio ScaranoMarina CaldaraAntónio Sebastião RodriguesMarina ScarpaAnuska V. AndjelkovicMarinos G. SotiropoulosAnvay UkidveMario CalveteAnzhelika VorobyevaMario GrassiAránzazu Zarzuelo CastañedaMarios SpanakisArellano María EvaristaMarisa ColoneAriana HuditaMarisol OcampoAristeidis ChiotellisMarité CardenasAristeidis DokoumetzidisMariusz MojzychArkadiusz JózefczakMariusz SkwarczynskiArkadiusz SurazynskiMarival BermejoArlene McDowellMariya SpasovaArnaud M. VigneronMariya VorobyevaArne SkerraMariza Abreu MirandaArokia Mariyadoss VijayanandMarjorie JonesArrigo CiceroMark AldersonArthur G. RobertsMárk BarokArtur GiełdońMark E. ByrneArtur ŚwierczekMark J. UlineArtur TurekMark MoloneyArzu AriMarko JukičAsta ZubrieneMarko KrstićAstrid SubriziMarko PavlovicAtheer AwadMarkus HofmannAtsushi FukushimaMarkus MesserliAtsushi KambayashiMarta GrochowiczAttila FarkasMarta KarazniewiczAura RusuMarta KepinskaAurélie SchoubbenMarta MarzecAurica P. ChiriacMarta OlejniczakAvinash KadamMarta PineiroAviva Shing Fung ChowMarta PlanasAvneesh KumarMarta PutrinšAxel HollmannMarta S. CarvalhoAxel T. NeffeMarta StrugaAyat A. AllamMarta SzekalskaAyca Yildiz-PekozMarta Ziegler-BorowskaAyman GradaMartin BauerBabatunde OkesolaMartin G. O’TooleBahareh AzimiMartin JaburekBahijja Raimi-AbrahamMartin KelloBalázs SarkadiMartin KucharBalla-Bartos CsillaMartin ŠímaBapan PramanikMartin SteupBarbara Blanco-FernandezMartin VlkBarbara ŁapińskaMartina PeršeBarbara MavroidiMartino Antonio DiBarbara MiziakMary Cano-SarabiaBarbara MognettiMaryam ArdalanBarbara R. ConwayMaryam NakhjavaniBarbara RóżalskaMary-Carmel KearneyBart KahrMarzena JamrógiewiczBartosz GrzeskowiakMarzio Luisa DiBasanth Babu EedaraMasahiro HiratsukaBastian BreustedtMasahiro ImamuraBeata NowakMasanobu KomatsuBeata StaniszMasanori HashimotoBeata Szulc-MusiołMasatoshi KarashimaBeatrice PerissuttiMassimiliano PerducaBegona Fonseca VázquezMassimo CarraroBegoña VerdejoMassimo LibraBei ChengMateusz DulskiBelen BeginesMats KarlssonBelén Begines RuizMatteo D’amoreBenedict LawMatteo ScampicchioBenedikt AsbachMatthew PanBenedito C. PrezotoMatthew T. WolfBenjamin LandMatthias WackerBeom-Jin LeeMattias PaulssonBernadette ByrneMaurice CollinsBernard HaendlerMaurizio MuracaBernat SoriaMauro BancheroBernd W. SiguschMauro ChinappiBernhard SchreflerMaxim PuchkovBertrand LiagreMaya ThanouBetül Arica YeginMayank Kumar SinghBharath Kumar VelMayank PatelBhasker RadaramMd Abdul HakimBianca GalateanuMd Abdus SubhanBianca NitzscheMd Jamal UddinBice ContiMd Masud ParvezBiliana NikolovaMeehyein KimBiljana BozinMehmet YilmazBiljana GlišićMehrez E. El-NagarBiljana MojsoskaMei-hwa LeeBlanca Elí Ocampo-GarcíaMeik NeufurthBłażej PoźniakMejía CarmenBlessing Atim AderibigbeMelani Anita SolomonBo LiMelánia BabincováBo SunMelchor Sanchez-MartinezBodnar RichardMelgardt M. De VilliersBogdan ParakhonskiyMelike UnerBoMi RyuMelissa SantiBongju KimMenotti RuvoBono LučićMeong Cheol ShinBoris BilcikMercedes FernandezBoris KrasnikovMercedes Fernández-ArévaloBoris KurganovMh Busra FauziBorislav AngelovMi Young LeeBorja Herrero De La ParteMichael A. FirerBozhao LiMichael ArkasBrad BarnettMichael BetteBranca Jacopo Junio ValerioMichael CangkramaBranka IvkovićMichael D. GrayBrecht VanbillemontMichael H. SmolenskyBrian E. LoveMichael JeltschBridgeen CallanMichael JuhnkeBrigitta LoretzMichael MorgenBritt WildemannMichael R. HamblinBruce AungstMichael R. KozlowskiBrullo ChiaraMichael RiederBruno Henrique VilsinskiMichael VogelbaumBruno Pereira CrulhasMichael WoeltjeBryan BeckinghamMichael Y. T. ChowBryan FullerMichał BiernackiBuddhadev LayekMichał BurdukiewiczBudimir MijovicMichal PecharButnariu, MonicaMichela PozzobonByoung Joon KoMichele De LucaByoung Soo KimMichele Francesco Maria SciaccaCaijun SunMichele Georges IssaCalogero FioricaMichele LanzaCamelia UngureanuMichele SavianoCameron AlexanderMichele SchlichCameron EvansMichelle E. FarkasCarla Marcella CaramellaMichiaki YamashitaCarla RizzoMick WellingCarlo GenoveseMicol Silic-BenussiCarlo IraceMieczyslaw PokorskiCarlos Eduardo Fonseca-AlvesMiguel A. PrietoCarlos GamazoMiguel Ángel PrietoCarly S. FilgueiraMiguel FerreiraCarmela SaturninoMiguel Gisbert-GarzaránCarmelo PugliaMiguel IdoateCarmen FerreroMiguel Mendez-RojasCarmen RinaldiMiguel SantosCarsten HerskindMiha LukšičCastro Whocely Victor DeMihaela Cristina BaicanCatalin LazarMihaela GheorghiuCatalin ZahariaMihaela Radu (Balas)Catalina Natalia Cheaburu YilmazMihai CenariuCatalina Valdés-BaizabalMihai MaresCatarina Roma-RodriguesMihalj PoŝaCatarina SeabraMihnea-Alexandru GamanCaterina GuiotMikhail M. Vorob’evCaterina VicidominiMikhail SolovievCC. Dennis WongMiklós PoórCélia R. CarliniMi-Kyeong JangCésar López-CamarilloMilad PourbaghiCesare DanesinoMilan TomaChaeho MoonMilena SorrentiChafia Touil-BoukoffaMilica MarkovicChai-Lin KaoMilica PantićChandrashekhar VoshavarMilica PešićChangqing SuMillán María Socorro EspuelasChao ZhangMilos KojicChao ZhaoMin Hua ChenCharles TruilletMing ChenChelladurai KaruppiahMing Hsien ChanChe-Ming Jack HuMing LiChen LingMing LuCheng-I. LeeMingzhou ChenCheng-Liang HuangMinjee KangChengyue WuMin-Soo KimChenshuang LiMiodrag JanicCheol MoonMira A.M.BehnamCheong-Weon ChoMira ParkCheuk Lun LiuMircea BeuranChi Bum AhnMircea Ioan PopaChia-Che HoMircea TampaChia-Hung LeeMireia MallandrichChiara Bianca Maria PlataniaMiriam Saiz-RodríguezChiara Emma CampiglioMirko ManettiChiara MazzoniMiroslava Dušková-SmrčkováChiara PosarelliMiroslava KacaniovaChiara RuzzaMiryam Criado-GonzalezChiara VitaleMiryana HémadiChiau Ming LongMirza BojićChie KojimaMititelu-Tartau LilianaChien-Ming HsiehMitja KolarChie-Shaan SuMitra AliabouzarChih-Li LinMitsutaka KitamuraChih-Sheng ChiangMladena Lalic-PopovicChi-Ming WongModestas ŽiliusChin-Chean WongMohamad AlamehChi-Ting HorngMohamed Abd ElkodousChris J. J. MulderMohamed Al-FatimiChristel NeutMohamed DarwishChristian AgatemorMohamed El-NabarawyChristian Erick PalavecinoMohamed M. Abdel-DaimChristian FercherMohamed W. AttwaChristian KastrupMohamed YoussryChristian MayerMohammad Abdul Motalib MominChristian ZurthMohammad FarhanChristina Cortez-JugoMohammad Javed AnsariChristina D. SotiropoulouMohammad M. Al-SaneaChristina KaravasiliMohammad MofidfarChristoph HiemkeMohammad NajlahChristoph PortierMohammad Tavakkoli YarakiChristopher CoteMohammad Zaki AhmadChulhun ParkMohan Kumar Muthu KaruppanChungChun WuMohankandhasamy RamasamyChunhong DongMohd Abul KalamChun-Ping JenMoises Armides Franco-MolinaChun-Ping LinMónica BenitoChunsheng GaoMonica M. JablonskiCinzia Anna VenturaMonica Mattioli Belmonte CimaCinzia PaganoMonica SalamoneCipriana StefanescuMonika Kadela-TomanekClare HoskinsMonirosadat SadatiClaudia AltamuraMoshe ShemeshClaudia ConteMoshi GesoClaudia KitzmüllerMostafa YazdimamaghaniClaudio BucoloMubashir HussainClaudio LuchiniMudasir Bashir GugjooClaudio N. CavasottoMuhammad MujtabaClaudio SalomonMuhammad Mustafa AbeerClaudio UcciferriMuhammad RasoolClive H. YenMuhammad Usman GhoriCodruta SoicaMuhammad ZafarCody PeerMuhhamad SajidColin BonduelleMulazim Hussain AsimColin C. SeatonMulenga BwalyaColin HareMunitta MuthanaColuccini CarmineMurali MaruthamuthuConcepció AmatMurali Mohan YallapuConcetta RafanielloMurtaza TambuwalaConstantin MircioiuMurugesan ChandrasekaranConstantin TamvakopoulosMusuc Adina MagdalenaCorral Inmaculada AranazMuzaffar IqbalCosima HirschbergMyoung-Hwan ParkCostas PanayiotouMyriam Van WinckelCrescenzio Francesco MinerviniMyron R. SzewczukCristian MateiNadir BettacheCristian Tudor MateaNadya G. GurskayaCristina ChircovNae Gyu KangCristina Maderuelo MartínNagendra Kumar KaushikCristina ManferdiniNageswara PilliCristina Manuela DrăgoiNanda MandavaCristina Mas-BarguesNan-Hung HsiehCristina Mihaela GhiciucNaoki UmemuraCristina Mihaela NicolescuNaoki YamamotoCristina NativiNaoko IkutaCristina PuigjanerNaoto TatewakiCristina-Maria TrandafirescuNarendar DudhipalaCristina-Mihaela LăcătușuNarmidi MamidiCristobal MirandaNasim NosoudiCrystal ShinNatalia A. MuralevaCynthia BosquillonNatalia KrastevaDae Hwan ShinNatália TomašovičováDagmar FischerNatalia V. BelosludtsevaDaiqin ChenNatalie TrevaskisDaisuke InoueNatallia V. DubashynskayaDaisuke MiyoshiNatasa MilosevicDaiva BironaitéNatassa PippaDale O. KiesewetterNatella EnukashvilyDalila MieleNathan LanningDana AkilbekovaNathan LazarusDana NiculaeNatividad Gómez-CerezoDandan GuoNavneet Kumar DubeyDanica JovićNebojša KladarDaniel Fernández-QuirozNebojša PavlovićDaniel FologeaNeel DesaiDaniel González-NietoNehal ElsherbinyDaniel H. González MaglioNela MalatestiDaniel KrugNelson FerreiraDaniel LiedtkeNemany Abdelhamid Nemany HanafyDaniel OttoNemany HanafyDaniel RekerNenad IgnjatovicDaniel Ricardo DelgadoNermeen Adel ElkasabgyDaniel RichterNesrein M. HashemDaniel SitarNéstor Mendoza-MuñozDaniel Xin ZhangNiccola FunelDaniela ArosioNicholas B. CarrigyDaniela CalinaNicholas FletcherDaniela IannazzoNicholas T. BelloDaniela MontesarchioNicola OreficeDaniela MontiNicolas SpinelliDaniele MassellaNicolas TaulierDaniele Roberto GiacobbeNicoleta RaduDaodao HuNidal JaradatDaria PodstawczykNiels SchaftDario RuscianoNikolay PolyakovDarja LavoginaNikunjkumar PatelDarya NovopashinaNina KurrleDavid G. CalatayudNina-Naomi KreisDavid GreenhalghNirmal MarasiniDavid MillsNisarg ModiDavid StepenskyNitesh K. KundaDavid TernantNitin PawarDavid VolkNitulescu MihaiDawid JanasNivedita ShettyDawiyat MassoudiNobuyuki MorimotoDe Gaulle I. ChigbuNoelia Caballero-Caserode Sousa Ângela Maria AlmeidaNoelia Sanchez-BallesterDebrati DasguptaNoelle S. WilliamsDelia Mihaela RaţăNohyun LeeDelia MunteanNora Suleiman-MartosDelicado Estrella NúñezNorbert RadacsiDelphine Chan-SengNoriaki Nagaiden Bergh Hubert VanNorifumi KawakamiDenis KainovNorival Alves Santos-FilhoDenis RychkovNoriyuki KajiDenitsa B. MomekovaNuno F. AzevedoDer-zen LiuNuno ValeDhananjay YadavNuria AlegretDharmendra K. YadavNuria Izquierdo-UserosDhaval K. ShahNuria SaperasDi ShiO. Andreea CojocaruDiana A. Van Riet-NalesOana CioancaDiana CoralloOana HosuDiana Díaz-GarcíaOana KarampelasDiana RafaelOctavian Mădălin BunoiuDianbao ZhangOfra BennyDibya PandaOkky Dwichandra PutraDidem Şen KaramanOl’Ga A. TarasovaDiego BayónOleg RevaDiego GolombekOlga D. ZakharovaDiego Romano PerinelliOlga LavrikDietmar KrautwurstOliver NeelsDijana JelićOliver StrbakDileep R. JanagamOliviu VostinaruDimitrios A. VrachatisOltea SampetreanDimitrios G. FatourosOluwatoyin A. AdelekeDimitrios TsiourvasOmar FahmyDimos D. MitsikostasOmar SarheedDinorah GambinoOndřej SlanařDionysia PapagiannopoulouOndrej VanekDipesh DhakalOrtensia Ilaria ParisiDirk H. OrtgiesOrtíz Héctor Iván MeléndezDivya SharmaOsmindo Rodrigues Pires JúniorDjordje MedarevicOsvaldo L. PodhajcerDmitriy ShurpikOvidiu OpreaDo Nam LeePablo TaboadaDolores R. SerranoPalaniyandi KaruppaiyaDolores Remedios SerranoPall EmokeDomenico MajolinoPálma FehérDominic Chih Cheng VoonPanagiotis BarbalexisDominique LesuissePanagiotis MallisDominique LunterPanita MaturavongsaditDonald TomaliaPankaj PandeyDonatella Degl’innocentiPanoraia SiafakaDonatella NegriPao-Chu WuDonatella VerbanacPaola MuggeoDong Hee NaPaola PelusoDong Hyun KimPaola Rosanna DiDongfang ZhouPaola SavoiaDongrim SeolPaolo CalicetiDong-Wook HanPaolo ColomboDoreen SchmidlPaolo GentileschiDorian MigońPaolo GiunchediDorina CoricovacPaolo MarianiDorota Gołąbek-SulejczakPapović SnežanaDorota MackiewiczParameswara Rao VuddandaDorota NeugebauerParthiban VenkatesanDorota Tichaczek-GoskaPascal OdouDorota Wójcik-PastuszkaPasqua Anthony J. DiDr. Julian RozenbergPasquale SimeoneDragana Mitić-ĆulafićPasqualina Liana ScognamiglioDragana VasiljevićPatricia BogdanovDriton VllasaliuPatricia Gálvez-MartínDu hyung ChoiPatricia Garcia-LopezDuo ZhangPatrícia SeverinoDušan RačkoPatricio Más-SerranoDwaipayan MukherjeePatricio Orellana-PalmaDzmitry ShcharbinPatrick M. GlassmanE. V. UspenskayaPatrick McardleEberhard UhlPatrizia Nadia HaniehEckart HanekePatrizia ZaramellaEdeildo JuniorPaul B. SavageEder RomeroPaul Chi Lui HoEdgardo DurantiniPaul D. BoudreauEdit CsapoPaul DalhaimerEdit FarkasPaul G. RoyallEdson Silva FilhoPaul JoyceEduardo Festozo VicentePaula BorrajoEduardo GuzmánPaula CoutinhoEduardo OliverPaula Malo De MolinaEduardo Padilla CamberosPaula OrtegaEdyta Gendaszewska-DarmachPaula SchaiquevichEishi AshiharaPaulius RuzgysElad MoisseievPaulo PaixãoElena A. RozhkovaPaulo Ricardo De SouzaElena ChekmenevaPaulo SantosElena DreassiPavel KopelElena I. RyabchikovaPavel RozsivalElena Maria VaroniPawan Kumar MishraElena MariottoPawel KrysinskiElena PeevaPawel NakielskiElena PopaPawel OlczykElena PurisPaweł PomastowskiElena RezusPedro Chamorro-PosadaElena SorokinaPedro PedrosaElena V. BatrakovaPegah VaraminiElena ZavyalovaPei-Li YaoElia BariPeixuan GuoEliana B. SoutoPengcheng WangEliana Dell’olmoPengxiang ZhuEliana LeoPensak JantrawutElisa PagninPer BäckmanElisa PanzariniPeta HarveyElisabete Pereira Dos SantosPetar PetrovElisabetta FanizzaPeter DurdikElisabetta SieniPeter F. KadorEliza WolskaPeter HagedornElka TouitouPeter HauserEllas SpyratouPeter O’ConnellEllen WasanPeter P. LinElsa GalbisPeter TimminsElwira SieniawskaPeter W. ThulstrupElzbieta Studzinska-SrokaPetr ZamostnyElżbieta WyskaPhilip Chi Lip KwokEman AshourPhilip GardinerEman AtefPhilip W. WertzEmanuel VamanuPhilipp OrekhovEmanuele GiurisatoPhilippe GarrigueEmanuele PapiniPia GiovannelliEmanuele PriolaPia Lopez-JornetEmerson Rodrigo Da SilvaPiaz Fabrizio DalEmilia SzymańskaPichet PraveschotinuntEmilio ParisiniPierangela TottaEmily H. PilkingtonPierfrancesco FrancoEmre ErdemPierfrancesco MorgantiEnrica ChiesaPierpaolo CeciEric GilPierre Eric JuifEric SundbergPietro AsproniErika AdomavičiūtėPietro ParcesepeErnani PintoPilar López-CornejoErnesto ReverchonPinalysa CosmaErsilia NigroPing YaoEryvaldo Socrates Tabosa Do EgitoPingping HanEugenio ProvenzanoPio ContiEuiseok LeePiotr BednarczykEun Jeong ChoPiotr CysewskiEva AndreuzziPiotr DobrzynskiEva BernalPiotr KurcokEva FenyvesiPiotr LadyzynskiEva NovotnáPiotr MichelEvandro WatanabePiotr PiszczekEvangelos AxiotisPiotr SzwedaEvelyn OrsoPo-Chang ChiangEven FossumPompilia Ispas-SzaboÉverton N. AlencarPoulami MajumderEvgenia Korzhikova-VlakhPradeep KumarEwa LaskowskaPradeep Kumar BollaEwa Stodolak-ZychPradeep Kumar SinghEwa TykarskaPrakash GangadaranEylon YavinPrakash SambharaEylul CetindagPrakash ThapaFabian StarsichPrasanna NeelakantanFabiana ConciatoriPrashant SharmaFabiana PicconiPrashanth NageshFábio Filipe Fereira GarrudoPriyadarshi ChakrabortyFabio Rocha FormigaPriyanka BhattFabrice PagniezPriyanka RayFabrizio BarberisPrzemysław DorożyńskiFabrizio MontecuccoPrzemysław KozminskiFabrizio SgolastraQian XuFaheem Hyder PottooQian YangFahim AhmadQin GaoFaiyaz ShakeelQingcheng MaoFaizul AzamRabijit DuttaFang LiuRachele CastaldoFarid RahimiRadek IndraFarshad Moradi KashkooliRadosław KowalskiFátima García-VillénRadu Claudiu FierascuFátima Milhano SantosRafael DavalosFazlurrahman KhanRafael GozalbesFederica FogacciRafael PeláezFederica RinaldiRafael Prado-GotorFederico Castro-MunozledoRaffaele CapassoFederico PercheRaid AlanyFédérico PercheRaimon SabatéFelipe VarumRainer DetschFelix RoyoRaisa N. KrasikovaFeng LinRaj KumarFeng-Lin YenRaj MukherjeeFengping DongRaj PaudelFerenc DomokiRajkumar BandiFerenc FenyvesiRajmohan DharmarajFernanda BoniRajni VermaFernando FerreiraRamireddy BommireddyFernando Notario-PérezRamon EritjaFernando PorcelliRania A.H. IshakFernando SotoRania HathoutFiona McCartneyRanjit DhengeFiorenza RancanRanjith KumavathFirdos Alam KhanRaphael MechoulamFlavia ZacconiRaquel De Melo BarbosaFlavio RizzolioRaquel Fernandez-GarciaFleming RodriguezRasangi WimalasingheFlora N. BalievaRaul MachadoFlorenci V. GonzálezRaymond J. LanzafameFlorentina GateaRebeca BustoFlorentina GolgoviciRegina Helena PiresFlorian C. GaertnerRegina KasperekFlorian M. WurmRémi RosièreFlorian SlimanoRemigiusz BąchorFlorin CiolacuRenata Puppin ZandonadiFlorin George HorhatRenata Salgado FernandesFotis IliopoulosRenato CuocoloFrancesc CanalsRicardo A.E. CastroFrancesca BorgnaRicardo LagoaFrancesca ButtiniRichard KeepFrancesca MastrottoRichard ParrishFrancesca RidiRichard UptonFrancesca S. ForiniRihani Sweilem Baseem AlFrancesca SciontiRita CortesiFrancesca SelminRita PatarrãoFrancesca UcchedduRitvij BowryFrancesco CacciolaRi-Yuan TangFrancesco CiccareseRóbert GáspárFrancesco Di GennaroRobert RoškarFrancesco GiansantiRobert StrickleyFrancesco GrossiRobert WojtyczkaFrancesco LodolaRoberta CazzolaFrancesco MaininiRoberta ImperatoreFrancesco MilanoRoberta SqueccoFrancesco StellatoRoberto Castro-MuñozFrancesco TavantiRoberto RonaFrancesco VisioliRoberto RozasFrancis BrakoRoberto Ruiz-CaroFrancisca RodriguesRobin CurtisFrancisco Alexandrino JúniorRobin TemmermanFrancisco AlvesRodica OlarFrancisco Fernandez-CamposRodolfo RomanachFrancisco Guillén-GrimaRoel DeckersFrancisco Javier Otero-EspinarRohit P. DugarFrancisco José OstosRoksana MarkiewiczFranciska ErdőRolf DanielsFranco BisceglieRoman B. LesykFranco CervellatiRoman LesykFranco D’OrazioRomana FatoFrançois-Xavier LegrandRomána ZelkóFrank BurcynskiRongcong LuoFrank KrauseRosa GaglioneFrank RosenauRosa Helena BustosFrédéric ChaubetRosa IacovinoFrédéric GobeauxRosales-Hernández Martha C.Frédéric TewesRosalia BertorelliFrederico PittellaRosalia Maria Cristina PellitteriFuat TopuzRosemeyre A. CordeiroFumitaka KogaRossella De MarcoFung-Fuh WongRossella SartoriusFu-Yin HsuRostyslav StoikaFuyuki SatoRuba IsmailGábor KatonaRudra PangeniGábor SomlyaiRuggero BettiniGabriel Aguirre-ÁlvarezRui VitorinoGabriel BatinRun ZhangGabriel EckermannRusso EleonoraGabriel Goetten De LimaRuth PrasslGabriel Herrero-BeaumontRyu ImamuraGabriela DudekS. G. KlochkovGabriela RapeanuSabine ZittaGabriela Romero UribeSabiruddin MirzaGabriela VochitaSabna KottaGabriele CarulloSabyasachi DashGabriele CaviglioliSachin ThakurGabriele CervinoSachiro KakinokiGabriele GrassiSadanand PandeyGabriele SadowskiSadri ZnaidiGabriella CsíkSaha PiuGaia BarisioneSahar EsfandyariGaia CodoloSahdeo PrasadGaia RocchittaSahi JasminderGajanan GhodakeSai H. S. BodduGanesan VaidyanathanSaikat BalaGarbine AguirreSaji UthamanGareth HealeySalim AlbukhatyGareth R. WilliamsSameh El SayedGarry WongSamet ÖzdemirGema Martinez-NavarreteSamir V. JenkinsGemma Di PompoSamuel SamnickGeorge KoliakosSamuel SilvestreGeorge S. KorresSana UllahGeorge SharbeenSandeep Kumar MishraGeorge Z. KyzasSandeep LohanGeorges VassauxSandeep UpadhyayGeorgi GeorgievSandile Phinda SongcaGeorgi MomekovSandra DavernGeorgia RagiaSandra G. ZárateGeorgios EleftheriadisSandra Oramas-RoyoGera SoniaSandra RistoriGerard Lee L. SeeSandra SimoesGerson J. RodriguesSandrine Cammas-MarionGert FrickerSang Gu KangGeta CârâcSangram SamalGhaith AljayyoussiSanja J. ArmakovicGhulam Md AshrafSanjuán Victor MangasGi Wook HwangSankar RenuGiacinto BagettaSanli MovafaghiGiacomo LuciSanta CirmiGiancarlo PascaliSantiago Daniel PalmaGianluca ViscusiSantiago Garcia-VallveGilberto AlvesSantiago Torrado-SantiagoGilberto RiveraSantina ChiechioGill DiamondSantosh BashyalGina MandaSara BernardiGiorgia MontalbandSara García-LinaresGiorgia UrbinatiSara Huerta-YépezGiorgio ColomboSara MoradiGiorgio FacchettiSara NavaGiorgio ManginoSara NicoliGiovanna CalabreseSara PerteghellaGiovanna LolloSara PintoGiovanna RassuSarbjit SinghGiovanna SotgiuSarika NamjoshiGiovanni AntoniniSarinj FattahGiovanni Codacci-PisanelliSasa VukmirovicGiovanni Luca RomanoSascha BeutelGiovanni MarascoSathish Sundar Dhilip KumarGiovanni SignoreSatoru KobayashiGirish PattappaSaurav Kumar JhaGiulia DonadelSayaka FujitaGiulia MagriScavello FrancescoGiulia SuaratoScott CanfieldGiulia VantiScott Derek BrownGiuseppe AlloattiSebastian DraackGiuseppe AmorusoSebastian GranicaGiuseppe CinelliSebastian HuthGiuseppe DionisioSebastian SchwamingerGiuseppe MartelliSebastiano TorrisiGiuseppe PaladiniSébastien BlanquerGiuseppe PeraleSebastien TaurinGiuseppe TripodoSekar VijayakumarGiuseppina IoeleSeong Hoon JeongGiuseppina NoccaSergei V. RaikGiuseppina SandriSergey M. AǐzikovichGiuseppina TommonaroSergio LiarteGleison Antônio CasagrandeSergio M. Granados-PrincipalGloria Huerta-AngelesSergio RecaldeGloria PerazzoliSergio ToddeGloria Pérez-RubioSergiu CoseriGodwin W. NchindaSeung Hwan YangGonzalo VillaverdeSeung Rim HwangGopinathan JanarthananSeyed Khosrow TayebatiGordana Wozniak-KnoppSezen VatanseverGoutam MondalShadab MdGraça SoveralShahid MansuriGraham LeggattShamroop Kumar MallelaGranero LuisShan ZhaoGrazia GalleriShankali PradhanGrazia MaugeriShanmugavel ChinnathambiGražyna Simha MartynkováShantanu SurGregor SersaShaohua JiangGregory PetersonSharan BobbalaGreta AdamczykSharareh Salar-BehzadiGreta FaccioSharida FakuraziGrzegorz BartoszSharif Md AbuzarGrzegorz CieslarShazid Md. SharkerGrzegorz CzernelSheba R. DavidGrzegorz NowaczykSheng YinGuang J. ChoiSheng-Min HsuGuan-Jhong HuangShengwen Calvin LiGuebum HanSherif T. S. HassanGuendalina ZuccariShigeru KawakamiGuenka PetrovaShih-Hsuan ChanGuilherme FerreiraShihori TanabeGuilherme M. GelfusoShinichi IkushiroGuillermo AhumadaShinji KobuchiGulam RabbaniShintaro FumotoGuohui LiShiouhwa JeeGuoju HongShiva GadilaGuru Raghavendra ValicherlaShixue WangGurudeeban SelvarajShoaib IqbalGuruprakash SubbiahdossShreesh SammiGustavo YannarelliShreyas KuddannayaGuy ZuberShrinivas VenkataramanGyörgy Tibor BaloghShuhua BaiGyörgy TrencsényiShui Yee LeungGyőző SzolnokyShuichi SetoguchiHaijun LiuShusuke TomoshigeHaisheng HeShyam Kumar GudeyHaishu LinSibel CetinelHamdy AbdelkaderSiddharth KesharwaniHamid MaadiSiddharth MatikondaHamid Reza HamediSiddharth S. KesharwaniHamidou KeitaSifei HanHamza Y. IsmailSilvana AlfeiHang QiSilvana FioritoHan-Joo MaengSilvia Alice LocarnoHanmant GaikwadSílvia Castro CoelhoHanna Dams-KozlowskaSilvia TampucciHanna V. BandarenkaSim NamkoongHannah BatchelorSimon DrescherHannah Katharine BatchelorSimon ŽakeljHany MareiSimona Liliana IconaruHao ChenSimona MartinottiHao YangSimona NeriHarald WodrichSimona SacchiniHarish HandralSimone AleandriHarleen KaurSimone Angela DeHarris PratsinisSimone BrogiHarsha KariyawasamSimone Di MiccoHasan RasouliSimonida TomićHassan GhonaimSin-Yeang TeowHassen AlmoazenSitansu Sekhar NandaHaya Lorberboum-GalskiSivakumar ManickamHeather BensonSiwang YuHea-Young ChoSlawomir MilewskiHee Eun KangSlobodan GadzuricHee Ho ParkSmita SalunkeHeiko HeerklotzSmruti ChaudhariHeinz BaumannSnežana D. SavićHelen E. TownleySnezana Ilic-StojanovicHelena BarrasaSofia A. PapadimitriouHelena KellySofia LimaHelena Margarida RibeiroSofia SoaresHemmen SabirSolange Teresinha CarpesHenrique FanecaSonal V. BhujbalHerbert HöpflSonali NashineHermann MansurSónia P. MiguelHermis IatrouSoojin JangHervé SchohnSophia GuHiba NatshehSorana BolboacaHidehiro UekusaSorin Marius AvramescuHidekazu KawashimaSoumyashree GangopadhyayHideo KatoSourav BhattacharjeeHidetaka NomaSouska ZandiHideyuki SatoSoyeun ParkHie-Won HannSpela ZupancicHilde Harb BuzzáSpiros VlahopoulosHimanshu KathuriaSpyridon PapageorgiouHimashu Narayan SinghSreeharsha NagarajaHira FatimaSreekanth VedagopuramHiroki TanakaSrinivasa Reddy BonamHiromi SakaiSrinivasulu AitipamulaHiromu KondoStanisław WołowiecHiroshi IkedaStanko SrcicHiroshi OmoteStavros MalamatarisHiroshi SugimotoStavroula NanakiHiroshi YagiSteen J. MadsenHirotaka MiyamotoStefan BroerHon Cheong SoStefan HainzlHongKee SahStefan ZwingenbergerHong-Ki LeeStefania MitolaHongwei ZhangStefania PetralitoHoon KimStefania SchiavoneHouwen PanStefania ZanettiHow TsengStefanie KleinHsin-Hui ShenStefano CardeaHsiu-Mei LinStefano FocaroliHsiu-Wen ChienStefano Girolamo PalazzoloHsueh-Te LeeStefano LeporattiHsueh-Wei ChangStefano PersanoHu HuangStefanos KikionisHuachun CuiStefanos RoumeliotisHuaiwen YangStephanie HehlgansHubert ZiółkowskiStephen B. CarrHugo Morales-RojasStephen ChimHugues J. ChapStephen InbrajHui JingStephen R. TomlinHui LiuSteve ArchibaldHui ZhaoSteven NisticòHui-Min WangSteven WillowsHumberto HernandezStrzemski MaciejHung-Wei YangStyliani KaranikaHung-Yi ChuangStyliani N. ChorianopoulouHuolong LiuSubramaniam JayanthiHusam YounesSubrata DebHussein N. RubaiySudip MondalHwan-Woo ParkSudip MukherjeeHwi-Yeol YunSue ChewHyang Moo LeeSue Hudson DuranHye One KimSultan B. DabagovHyeun Joong YoonSundeep SinghHyo-Eon JinSupandeep Singh HallanHyuk Sang YooSuping LiHyunah ChoSurendra NimeshHyun-Jong ChoSurinder SoondI. M. Le-DeygenSusana Diaz-AmayaIan C. FrancisSusanna GuernelliIan KerrSusumu UchiyamaIan S. BlagbroughSven SchnichelsIan Steuart MillerSvetlana IbrićIan WymanSvetlana JovanovićIbrahim BitarSvetlana TamkovichIchiro MaruyamaSvetoslav TodorovIeva PlikusieneSwayam PrabhaIgor E. DeyevSyed A. QuadriIgor V. KhudyakovSyed ImamIkram Ullah KhanSyed Sarim ImamIl Soo MoonSyn YeoIlaria Dal PráTabito KinoIlaria Elena PalamàTadakazu KondoIlaria OttonelliTae Joon KwakIlaria RubinoTae-Gyu LimIldikó BácskayTae-Jin KimIldikó CsókaTaesup LeeIleana RauTaher HatahetIlya ShenderovichTahseen H. NastiIm Sook SongTaiki MiyazawaIman KavianiniaTaís GratieriIman YazdiTakanori GoiImran HouseTakashi MatsuwakiImran KazmiTakayuki FuruishiImtiaz KhanTakehisa HanawaInbaraj Baskaran StephenTakenori HarunaInchan KwonTakeshi OmasaInese FilipovaTakeshi ShimomuraIngrid BurvenichTakumi IshizukaIn-hwan BaekTamara I. PajpanovaInnocence HarveyTânia SilvaIn-Soo YoonTanja IlićIoana ChiulanTanveer Ahmad TabishIoana Cristina OlariuTao YangIoana Dorina VlaicuTao YiIoana MozosTao ZhangIoannis NikolakakisTapan NayakIoannis PartheniadisTaro ShimizuIoannis PirmettisTatiana Souza PortoIolanda De MarcoTatiana V. PletenevaIosif MarincuTatsuaki TagamiIra. KatzTatsuhiko SatoIrati RodrigoTatsuya UsuiIrena NalepaTatyana O. PleshakovaIrena Wojsyk-BanaszakTatyana ShabatinaIrina NegutTatyana V. VolkovaIrini DoytchinovaTe I. LiuIsabel Gonzalez-AlvarezTejabhiram YadavalliIsabel Sánchez PérezTeresa CastilloIsabella PanfoliTeresa GarriguesIsha TanejaTeresa M. R. MariaIsmaiel TekkoTeresa MusumeciIsrafil KucukTeresa Simon-YarzaIstvan AntalTeresa TrottaIstván AntalTero JarvinenIstván CsontosTeruki NiiItalia FalconeTeruo MurakamiItzik CooperTetsumi IrieIvan DimitrovTetsuya MiyanoIvana CacciatoreThais AlvesIvana D’AngeloThanh Kha PhanIvana Savic GajicThashree MarimuthuIvelina TsachevaThazha P. PrakashIveta BernatovaThiagarajan MadheswaranIvonne Bazwinsky-WutschkeThierry VandammeIwona GabrielThomas GrothIwona GolonkaThomas HellwegIzabella Karska‐BastaThomas RuppertIztok GrabnarThomas S. GriffithJacek GębickiTianlong LiJacopo BurrelloTianqing LiuJacqueline KentTibor HianikJae Hwan JungTibor NagyJagadeesh K. VenkatesanTim MaischJagdeep K. SandhuTim SmithJagdish Kumar JaiswalTimothy HeathJagoda LitowczenkoTimothy O. AdekoyaJain PriteshTimur SalievJakub Dalibor RybkaTina KaussJakub SzlękTina McKayJakubczyk EwaTinchun ChuJames A. AngusTitus VlaseJames B. FinkTiziana EspositoJames S. MeabonTofayel AhmedJamil Awad ShibliTomasz BujakJan Henrik FinkeTomasz GoszczyńskiJan MacutkevicTomasz GrabowskiJan SteenekampTomasz RogozińskiJan ZitkoTomislav MeštrovićJana GevertzTommaso FelicettiJana HeldTommaso TabaglioJanja StergarTomofumi YamamotoJann SarkariaTomohiko SasaseJanthima MethaneethornTomohiro OsakiJaqueline Carvalho De OliveiraTomoki MaedaJarkko RautioTomomi TakanoJarmila JedelskáTong DengJaroslav TruksaTony K. L. KiangJasmina VidicTony Le GallJason LiToshi NagataJason T. McConvilleToshihiko TashimaJason Thomas DuskeyToshihiro KushibikiJavad TavakoliToshio TakahashiJaved AhmadToshiyuki KawaiJavier MoralesToshiyuki SakaedaJavier Octavio MoralesToyofumi SuzukiJavier P. GisbertTracy A. StoneJavier Rodríguez VillanuevaTruong Phuoc NghiaJavier Ruiz-EderraTsung-Jen WangJavier Sánchez-NievesTsvetelina VelikovaJavier UserosTuo MengJay MishraTushar KumeriaJayachandran VenkatesanU Gianfranco SpizzirriJean-Claude DaranUdo BakowskyJean-Daniel MalcorUgo D’AmoraJean-Luc PoyetUlf AndereggJean-Marc MalingeUlrich HackerJee-Heon JeongUmberto MusazziJeffrey M. TingUrszula BazylinskaJelena DjurisUrszula NawrotJelena M. JanjicUrszula StachewiczJelena ParojčićUrszula ZarzeckaJelena PoljarevicUwe SteinhoffJeng-Shiung JanVadim Le JoncourJennifer FiegelVadim PokrovskyJennifer GubitosaVadim YansholeJennifer HulingVaishali KapoorJennifer PattersnValentin JercaJennifer PattersonValentin ReutovJenny Ka Wing LamValentina RubinoJens KrügerValentina UivarosiJer-An LinValeria LallaiJerzy ChudekValério Monteiro-NetoJessica Dal ColValeriu Marin ȘurlinJessica M. AndrioloValvanera Vozmediano EstebanJesús Fernández-LucasVan Tu DuongJesús M. SanzVanderlei Salvador BagnatoJesus Olivero-VerbelVanessa Carla Furtado MosqueiraJiafen HuVangelis KaralisJiajia XueVanja TadićJiali ZhaiVasanth KumarJian LingVassiliki PapadimitriouJiangeng HuangVenerando PistaràJiao Jiao LiVenkata MachhaJiaxiang ZhangVeronika HuntosovaJiayi PanVeronika KhairullinaJim BallingerVeronika PavliňákováJin GaoVeronique PreatJingjing GaoVesna VasicJingjing SunVicent RodillaJingquan WangVictor RevinJinming HuVictoria LeiroJinsong HaoVictoria O. ShipunovaJinxiang XiVijay Kumar ThakurJin-Yi WuVijay Sagar MadamsettyJiri BarekVijayalaxmi G. GuptaJiwei GuVi-Khanh TruongJoachim GeyerViktor Korzhikov-VlakhJoachim StorsbergVillasante Alberto OuroJoan OlivaVincenzo De LeoJoana AntunesVioleta PopescuJoana BickerViorel NastasaJoana LoureiroViorica MusatJoana MagalhãesVishnu Priya MuraliJoana ValenteVišnja StepanićJoanna DepciuchVitalis BriedisJoanna KarbowniczekVivek TrivediJoanna Skopinska-WisniewskaViviana De CaroJoanna Stanislawiak-RudowiczVladimir A. TimoshevskiyJoanna WietrzykVladimir DobričićJoao MamedeVladimir DyakonovJoão Paulo LealVladimir Eugenievich YudinJoão SilvaVladimir M. Gun’koJoaquim VivesVladimir V. ZarubaevJoaquín C. García-MartinezVolker SchirrmacherJoaquín María Campos RosaWai Yee YeongJochen StrubeWaldemar MrózJochen UtikalWalkiria SchlindweinJoelle SobczakWayne ThomasJohan GisingWei ChenJohannes NotniWei Chien HuangJohn B. MorrisWei HuangJohn MackWei Long NgJohn S. SvendsenWei ShaoJoias HammanWei ZouJoji OkazakiWei-Chao LinJoke Den HaanWeien YuanJoke M. M. Den HaanWeifeng LinJonas ContieroWeihua ZhouJonathan O’Keeffe AhernWeiling YuJong Bong LeeWeilue HeJongbum SeoWeimin CaiJongho JeonWeixiong LiangJong-Seok ParkWenjing LinJong-Suep BaekWerner WeitschiesJooho ParkWesam S. ShehabJörg BreitkreutzWing-Fu LaiJorge Góngora-RodríguezWing-Hin LeeJorge SantosWitold JakubowskiJosé A. MartinsWitold JamrózJose BernaWladyslaw WeglarzJosè Divino-FilhoWolfgang W. LeitnerJosé Juan Ordaz-OrtizWolf-Rainer AbrahamJosé L. Jiménez BlancoWooram ParkJosé Pérez-RigueiroWujie ZhangJosé R. PinedaXi ZhaoJose Ramon Moyano-MendezXian LuoJoseph LovecchioXiaochun WanJoseph OrtegaXiaohong WangJosy A. OsajimaXiaohu HuangJosy Anteveli OsajimaXiao-Jia ChenJosy AugustineXiaoqing HuaJozef Al-GousousXiaowen HuJózsef TőzsérXiaozhong QuJr-Jiun LiouXijun PiaoJu CarmonaXi-Lu ChenJu Hee RyuXin ChenJuan BarcenaXin SunJuan Bautista De SanctisXinchang ZhangJuan GalloXingmin ZhangJuan Luis ParisXiuhua ZhaoJuan Manuel AragonesesXudong CaoJuan Pablo RigalliXue LiuJuan ParisYahya E. ChoonaraJuan PellicoYajun WangJuan Pérez-FernándezYan ShuJuan Rubio AlonsoYang LiJuan TorradoYang LiuJuan VillenaYan-Hsiung WangJuan ZhaoYanis Toledano-MagañaJuei-Tang ChengYan-Ming XuJui-Chen TsaiYannis KaramanosJulia Fernández-PérezYao-Chang ChiangJúlia M. C. S. MagalhãesYasuko ObataJuliann M. Di FioreYasushi AranoJulien Dugal-TessierYasuyuki GotoJulien RossignolYaswanth KuthatiJulien Saint-PolYayoi KawanoJulio AlarconYeon Sun LeeJulita KulbackaYeu SuJun ToyoharaYi LuJun WeiYi-Chen ChenJun XuYi-Cheng HoJung-Woo BaeYifeng CaoJung-Woo ChaeYihenew Simegniew BirhanJun-Ichi KadokawaYit Lung KhungJun-Jen LiuYi-Ting ChiangJunjiang SunYiwei TianJunjie LiYlenia ZambitoJun-O. JinYohann CorvisJunzi WuYoichi SakuradaJuraj MajtanYoichi ShimizuJya-Wei ChengYongfeng GaoJyh-Fei LiaoYonggang PeiJyothsna ManikkathYongjun SuiKa L. HongYongxiang LuoKai GriebenowYoon-Jee ChaeKai-Wen HsuYoshiaki HiranoKállai NikolettYoshihiro NoguchiKamalinder K. SinghYoshikazu NakamuraKamil ÜneyYoshikuni TeramotoKamila PolgarovaYoshio TakesueKamila Sroda-PomianekYoshiyuki MishimaKandasamy SaravanakumarYoshiyuki ShirasakaKapil D. PatelYoshiyuki TatsumiKapil PatelYoung Bong KimKaren M. ScanlonYoung Whan ChoiKaren MethlingYousuf MohammedKarin HufnaglYou-Zhi TangKarl HemmingYu Chul KimKarla Juarez-MorenoYu I. DeryabinaKarol P. NartowskiYu SunKarol SteckiewiczYu ZhangKarola Vorauer-UhlYuanjing LinKarolina Skonieczna-ŻydeckaYub Raj NeupaneKarsten KnoblochYu-Fang ShenKarsten KöhlerYufeng ZhouKarthik NagapudiYuichiro HatanoKasturi Joshi-NavareYuichiro KobayashiKatalin MonostoryYuichiro NoiriKatalin Réti-NagyYujiao LuKatalin UrayYun LingKatarina NikolicYung-Chuan LiuKatarína ValachováYunjin JungKatarzyna DettlaffYuri DancikKatarzyna JaszczYurij StetsyshynKatarzyna Kosicka-NoworzyńYury A. SkorikKatarzyna KotfisYusuke AzumaKatarzyna LewandowskaYusuke HattoriKatarzyna RoszekYusuke HirataKatarzyna SosnowskaYusuke SatoKatarzyna Sykłowska-BaranekYutaka InoueKatarzyna Zabielska-KoczywąsZahari VinarovKateřina PetříčkováZahra El-SchichKatharigatta VenugopalaZalba SaraKatherine MargulisŽarko MitićKathia M. HonorioZdenek KejikKathleen A. R. SchildrothZebunnissa RamtoolaKatrina AlbertZeeshan AhmadKatrina MirandaZeid A. NimaKaushalendra ChaturvediZeineb AturkiKavindra Kumar KesariZeneng ChengKay D. BeharryZengqian ShiKay Dietrich WagnerZhaohai QinKazuhiko MatsuoZhe YangKazuhisa MatsunagaZhenghong LeeKazuma OgawaZheng-Rong LuKeerti JainZhifeng DuKeitaro SouZhijun WangKeith HorspoolZhijun YangKeith RochfortZhixia LiuKelly A. KimmerlingZhongwei LiuKelly Johana Figueroa LópezZhou QiKen LeungZili SideratouKenneth HettieZiwen JiangKenneth P. MittonZiying YanKênnia Rocha RezendeZofia Urbanczyk-LipkowskaKentaro YoshidaZoltán JakusKeon Wook KangZoltán KovácsKeshav GopalZoltán VerébKetan M. RanchZoltán WienerKeumhan NohZoran ZekovićKeun-Sik KimZorana MilanovicKevin A. FenixZoubir DjeradaKevin LiZrinka RajićKevin McHughZsolt GállKevin MontagneZvetanka ZhivkovaKevin NashZvezdelina Lyubenova YanevaKhalid Mohamed El-Say



